# Rheological and Chemical Effects of Waste Tire Pyrolytic Oil and Its Encapsulation as Rejuvenators on Asphalt Binders

**DOI:** 10.3390/polym17182449

**Published:** 2025-09-10

**Authors:** Rodrigo Delgadillo, Araceli González, Ixa Marzal, Jose L. Concha, Cristina Segura, Luis E. Arteaga-Pérez, Jose Norambuena-Contreras

**Affiliations:** 1 Departamento de Obras Civiles, Universidad Técnica Federico Santa María, Valparaíso 2390123, Chile; araceli.gonzalez.12@sansano.usm.cl (A.G.); ixa.marzal.12@sansano.usm.cl (I.M.); 2 LabMAT, Departamento de Ingeniería Civil y Ambiental, Facultad de Ingeniería, Universidad del Bío-Bío, Concepción 4051381, Chile; jlconcha@ubiobio.cl; 3 Unidad de Desarrollo Tecnológico, Universidad de Concepción, Coronel 4191996, Chile; c.segura@udt.cl; 4 Department of Chemical Engineering, Faculty of Engineering, Universidad de Concepcion, Concepción 4030000, Chile; larteaga@udec.cl; 5 Advanced Bituminous Materials Laboratory, Department of Civil Engineering, Faculty of Science and Engineering, Swansea University, Bay Campus, Swansea SA1 8EN, UK

**Keywords:** rejuvenator, asphalt, encapsulation, recycling, waste tires, pyrolysis

## Abstract

This study investigates the rheological and chemical effects of waste tire pyrolytic oil (TPO) and its encapsulation (POC) as rejuvenators for asphalt binders. Driven by the need for sustainable and effective strategies to Recycle Reclaimed Asphalt Pavement (RAP), we investigated the use of TPO in two forms: as a liquid additive and as polymer capsules. The capsules, made in a 1:5 mass ratio (one part polymer, five parts TPO), were assessed through two methods: rheological tests (dynamic modulus and phase angles) and chemical composition analysis (carbonyl and sulfoxide indices). The binders underwent three aging levels: unaged, primary aging (RTFO), and secondary aging (PAV). Five liquid TPO dosages (1%, 2%, 4%, 6%, 9% by weight) and three encapsulated TPO dosages (6%, 9%, 12% by weight) were tested. Results show that TPO reduces stiffness, increases viscous response, and lowers aging indices, with higher dosages enhancing the effect. Quantitatively, 9% liquid TPO restores PAV-aged binder to near-unaged conditions, suitable for RAP recycling, while 4% release from POCs achieves rejuvenation comparable to RTFO-aged binders, enabling self-healing applications. The estimated release of TPO from POCs during mixing was 20–40%, ensuring a gradual softening effect. These findings highlight the potential of TPO and POC in enhancing asphalt durability and recycling.

## 1. Introduction

Asphalt mixtures represent one of the most extensively utilized materials for road paving applications. In 2021, the United States and the European Union produced about 392 million tons and 291 million tons of asphalt mixtures, respectively [1]. The production of asphalt mixtures requires significant energy inputs, primarily reliant on oil-derived materials such as asphalt binders and mineral aggregates. As a result, the life cycle involved in the construction, operation, and maintenance of paved roads leads to significant environmental impacts [2]. Given these concerns, there is an urgent need to integrate recycled asphalt mixtures and adopt more sustainable feedstocks in road infrastructure construction [2].

One of the prevalent strategies in integrating sustainable material practices within road infrastructure is the utilization of Reclaimed Asphalt Pavements (RAPs). Although the use of RAP is technically feasible, the recycled material frequently fails to maintain the mechanical and chemical properties of the original asphalt due to the inevitable deterioration it undergoes during its service life. This damage, commonly referred to as asphalt aging, involves both primary and secondary aging processes [3]. Primary aging occurs during asphalt mixture production and pavement construction, where exposure to high temperatures and oxygen accelerates the degradation of the binder. Conversely, secondary aging occurs throughout the operational lifespan of the pavement, characterized by the gradual oxidation of the binder induced by factors such as UV radiation, moisture, atmospheric conditions, and variations in traffic loads [3].

At the nanoscale level, asphalt binders are colloidal systems comprising insoluble asphaltenes dispersed in maltenes. Aging induces chemical structural alterations in the binder, including the formation of new functional groups (e.g., C=O and S=O). Interactions among these groups and other components in the binder structure lead to asphaltene aggregation, which increases the asphaltene-to-maltene ratio in the mixture. These molecular changes also affect the mechanical performance of the binder by increasing its stiffness, promoting microcrack formation, and compromising pavement integrity [3,4].

In this context, several studies have emphasized the importance of using chemical additives such as rejuvenators to restore the mechanical performance of aged asphalt mixtures [5,6,7,8,9]. Rejuvenators are extender oils with a high proportion of maltenes, which upon addition to the aged binder, recover its asphaltene-to-maltene ratio. Consequently, rejuvenators facilitate the re-dispersion of the asphaltenes in the colloidal system, promoting a physicochemical restoration of the asphalt mixture [10,11].

Rejuvenators can be obtained from different sources, such as crude oil, vegetable oils, waste oils or pyro-oils from biomass and waste tires [6]. Recently, the use of waste tire pyrolytic oils (TPOs) as fuels, chemical additives and asphalt rejuvenators has drawn great attention [12]. Norambuena-Contreras et al. suggest that the high proportion of maltenes (<90% wt.), low viscosity (<10 cP) and density of the pyro-oils (<800 kg/m^3^) of TPOs explains their effectiveness in restoring microcracks in aged asphalt mixtures [13]. In a later study, Quezada et al. combined spatio-temporal experiments with full-atomistic molecular dynamics (MD) simulations, showing that TPO can diffuse into aged bitumen, reducing its viscosity and promoting self-healing of microcracks [14]. Kumar et al. [15] studied the chemical and rheological effects of three asphalt binders and found that adding 9% waste tire pyrolysis oil to aged PAV binders restored their properties to levels similar to the original binder.

Although TPO shows promising potential as an asphalt rejuvenator, its effectiveness may be influenced by factors such as its chemical composition, the dosage added to the aged binder, and the application technology used. There are three methods for applying rejuvenating treatments in asphalt mixtures: (i) surface rejuvenation, where TPO is directly added to the damaged pavement surface, (ii) using TPO as an additive during RAP mixture preparation, and (iii) using Pyrolytic Oil Capsules (POCs) to promote extrinsic self-healing in asphalt mixtures. These applications have been well-documented in previous literature, with a consensus that, regardless of the method, the rejuvenator must diffuse into the damaged asphalt binder for effective rejuvenation [16,17,18,19,20].

Furthermore, the dose of rejuvenator used for surface, RAP or encapsulated treatments is different as the degree of the required restoration varies among them. For example, when using RAP, its properties must be restored to conditions similar to new asphalt [5]. The extensive rejuvenation required for RAP is because the rejuvenated material must undergo primary aging during the production and construction process [21]. In contrast, the encapsulated rejuvenator would be released from the capsules into the asphalt pavement after construction. Therefore, the required degree of rejuvenation in this case is similar to that of a primary aged state [22,23]. Conversely, excessive amounts of rejuvenator can soften the mix, leading to permanent deformation of the pavement [24,25].

These changes can be monitored by studying the rheological properties of the binder, such as the dynamic modulus (|G*|) and the phase angle (δ) [3,4]. The softening effect of the rejuvenators will produce a decrease in the |G*|of the aged binder, while increasing the δ of the aged material due to the restoration of the viscous component of the material [26,27]. In addition, the rejuvenation effects can be chemically tracked by studying structural transformations using Fourier Transform Infrared spectroscopy (FTIR). This technique is widely used to detect chemical changes in asphalt mixtures by monitoring IR signals associated with carbonyl (1700 cm^−1^), sulfoxide (1030 cm^−1^), and aromatics (1600 cm^−1^). The literature agrees that the relative quantity of these functional groups (defined as indexes) should decrease when the binder is rejuvenated [28,29,30]. Nevertheless, several research gaps remain unaddressed, including (i) the influence of TPO and POC dosage on the chemical properties and rheological behavior of aged asphalt binders, and (ii) the activation level of POCs in aged asphalt binders under different rheological conditions for rejuvenation purposes.

In conclusion, the effectiveness of TPOs as asphalt rejuvenators is influenced by factors such as chemical composition, dosage, and application method. Therefore, integrating rheological and chemical characterizations is crucial to understanding the chemo-mechanical mechanisms behind the self-healing process. This study systematically evaluates the rejuvenating effects of TPOs on binders with varying aging levels and, for the first time in the literature, compares two application methods: liquid TPO and encapsulated POCs. We assess binder rheological properties (|G*|, δ) and chemical composition using FTIR analysis. These analyses offer valuable insights into the effectiveness of TPO and POCs, supporting the design of more efficient and sustainable asphalt pavements.

## 2. Materials and Methods

### 2.1. Asphalt Binder and Additives Based on Pyrolytic Oil

**Figure 1:**
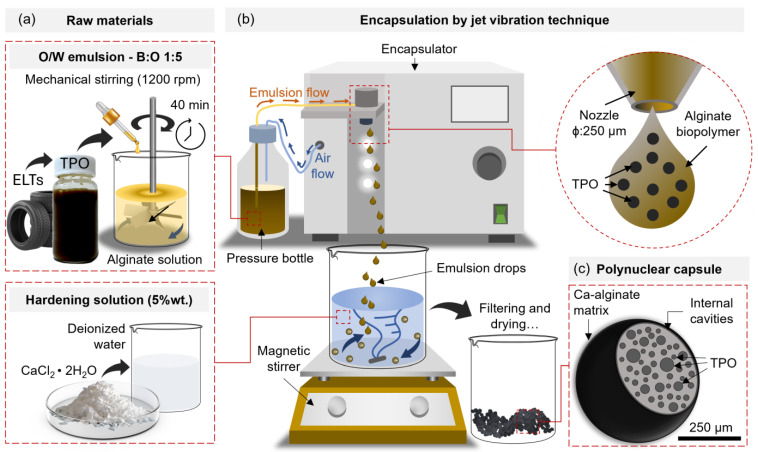
Encapsulation of TPO in bio-based polymeric capsules, describing (**a**) the raw materials used, (**b**) the encapsulation through the jet vibration technique, and (**c**) the capsule morphology.

The asphalt binder used in this study is an unmodified binder with a Superpave PG64-22 classification [31]. The main properties of this asphalt binder are summarized in Table 1. Additionally, the additives for the asphalt binder include Tire Pyrolytic Oil (TPO), which was obtained from the pyrolysis of waste tires, and Pyrolytic Oil Capsules (POCs), produced by encapsulating TPO using jet vibration technology (see Figure 1).

### 2.2. Evaluation of the Tire Pyrolytic Oil (TPO) and Pyrolytic Oil Capsules (POCs)

The TPO was obtained from the pyrolysis of waste tires. Prior to the reaction, the waste tires were crushed, and the steel, textiles and other non-polymeric materials were separated from the mixture. Thereafter, the polymers (granules < 1 cm) were subjected to pyrolysis in a stirred reactor at 450 °C, 2 h dwell time, 50 °C/min heating ramp, and TPO was recovered by condensation at 15 °C. More details on the pyrolysis facility and procedures can be gathered from previously published papers [14]. The mass yield (mass of TPO/mass of waste tires) of TPO was between 40 and 44% wt. and it featured a density of 0.89 g/cm^3^, viscosity 7.89 mPa∙s @20 °C, a pH of 6.27 @25 °C and <3% wt. water content. A full characterization of the TPO, including FTIR and gas chromatography coupled with mass spectrometry (GC-MS), was reported in a previous paper by Quezada et al. [14]. The TPO was used in the rejuvenation test in both liquid and encapsulated formats: (i) as liquid to simulate surface and RAP rejuvenation and (ii) as encapsulated rejuvenator to simulate an asphalt self-healing application.

The Pyrolytic Oil Capsules (POCs) were synthesized from a low-viscosity sodium alginate biopolymer (viscosity ≤ 300 mPa∙s, density 1.02 g/cm^3^ in a 2% wt. solution) and calcium chloride (CaCl_2_) dihydrate (77% purity), using the jet vibration method reported by Concha et al. and shown in Figure 1 [23]. First, an Oil-in-Water (O/W) emulsion was prepared by mechanical agitation, where the TPO was added into the alginate solution using a biopolymer:oil (B:O) mass ratio of 1:5. Then, the emulsion was extruded through an encapsulator device, separated into droplets and collected in a 5% wt. CaCl_2_ solution. As a result, the POCs presented a polynuclear internal morphology, where the TPO was distributed across multiple internal cavities of the alginate biopolymeric structure. Based on the test characterization methods described in [23], the capsules presented an average size (diameter) of 397 μm and an encapsulation efficiency of 92.51%. The capsule size was selected to ensure efficient distribution within the bitumen sample and to remain smaller than the gap between the plates used in rheological tests.

### 2.3. Aging Process of Asphalt Binders

Bitumen PG64-22 was aged using a standardized Rolling Thin Film Oven (RTFO) to simulate short-term aging during asphalt mix production and pavement construction. The RTFO-aged binder then underwent Pressure Aging Vessel (PAV) tests according to ASTM D 6521-13 to simulate long-term aging that occurs during the binder’s in-service life within the asphalt pavement [32].

### 2.4. Mixing of Asphalt Binder with TPO and POCs

The PAV-aged binder was mixed with TPO in several doses according to the methodology published by Concha et al. [33]. In a typical experiment, 30 g of PAV asphalt binder was poured into a 100 mL Pyrex glass beaker and heated to 140 °C on a hot plate, being mechanically agitated at 300 rpm for 20 min. During this process, the TPO was incorporated dropwise at 5 different contents: 1%, 2%, 4%, 6%, and 9% wt. of PAV binder. Similarly, for the POCs, the same mixing steps were followed. However, in this case, the capsules containing TPO were incorporated into the PAV binder at three different concentrations: 6%, 9%, and 12% wt. of PAV binder. The doses of capsules were corrected by considering the encapsulation efficiency estimated during their synthesis (i.e., mass of TPO within the capsule/mass of TPO in the emulsion = 0.83).

Finally, samples with liquid TPO were named PAV-TPOi, with “i” meaning the percentage of TPO by weight of the binder. In the same way, test samples with capsules were named PAV-POCi, with “i” meaning the percentage of POC by weight of the binder. The binder samples without additives in the unaged, primary aged and secondary aged states were named unaged, RTFO, and PAV, respectively.

### 2.5. Rheological Characterization of Aged Asphalt Binders with TPO and POCs

The rheological characterization was carried out with frequency and temperature sweep tests [34,35]. A Dynamic Shear Rheometer (DSR), Anton Paar model MCR 301, with Anton Paar RheoCompass^®^ software (version 1.30.1164) was used. The test frequencies were varied between 100 and 0.1 rad/s and the temperatures between 5 and 75 °C. The maximum shear strain applied in all test conditions was 0.5%. Plates with 8 mm diameter and 2 mm gap and plates with 25 mm diameter and 1 mm gap were used, depending on the aging level and the testing temperature of the sample. Samples with capsules (POCs) were tested using 8 mm diameter/2 mm gap plates, since the capsule size (397 µm) required a gap at least 4 times larger for ensuring an accurate characterization of the two-phase material [36,37]. The tests were performed according to ASTM D7175 [36]. The master curves of |G*| and δ variables were constructed for each material, using the time-temperature superposition principle described by Papagiannakis and Masad [38]. The reference temperature selected for the master curves was 25 °C, aligning with the intermediate temperature for a PG 64-22 binder.

### 2.6. Characterization of Asphalt Binders by Means of FTIR-ATR

All the recorded spectra were normalized to reduce any difference related to the beam penetration and avoid any bias in the further interpretation of the results. In addition, chemical changes produced by the addition of TPO and POCs were analyzed using spectroscopic-based descriptors of Pipintakos et al., as follows [39]:

(1) $$I_{C=O}\;=\;\frac{A_{1700\;}}{\sum_{i=0}^{n}A_{n}}:\;Carbonyl\;index$$

(2) $$I_{S=O}=\frac{A_{1030\;}}{\sum_{i=0}^{n}A_{n}}:\;Sulfoxide\;index$$

(3) $$I_{\mathrm{Comb}}={\;I}_{C=O}+I_{S=O}:\;Combined\;index$$

where n = 724, 743, 814, 864, 1030, 1376, 1460, 1600, 1799, 2862, 2953 cm^−1^.

**Figure 2:**
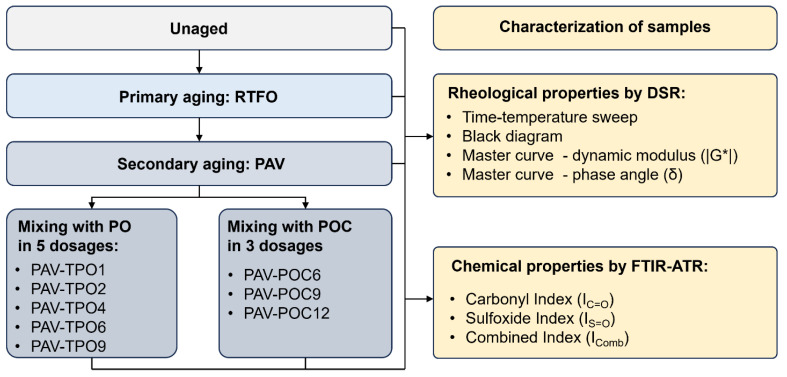
Experimental plan applied to the analysis of chemical and rheological changes induced in aged asphalts modified with TPO and POCs.

Additionally, a detailed description of the experimental plan is summarized in Figure 2.

## 3. Results and Discussion

### 3.1. Rheological Measurements

**Figure 3:**
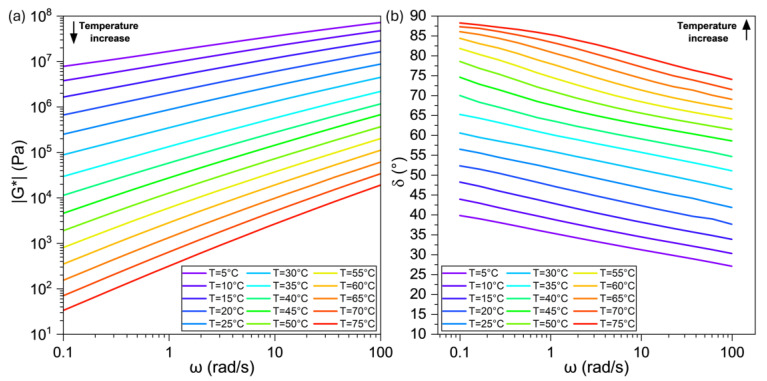
(**a**) |G*| and (**b**) δ curves for the PAV-TPO4 material at different frequencies and temperatures.

The rheological parameters dynamic modulus (|G*|) and phase angle (δ) represent the stiffness and elasticity of the asphalt, respectively. A higher |G*| value signifies a stiffer material. A lower δ value represents a behavior that is more elastic and less viscous. The expected response for asphalt binders subjected to frequency and temperature sweep tests is as follows: when the temperature is increased and when the frequency is diminished, the value of |G*| decreases; when the temperature is raised and when the frequency is diminished, the value of δ increases. For all tested specimens containing TPO in its liquid and encapsulated form (POC), the results of the frequency and temperature sweep tests showed the trend expected for asphalt binders, indicating a compatible rheological behavior of the TPO with the asphalt binder. As an example, the |G*| and δ results for the PAV-TPO4 sample are presented in Figure 3a and Figure 3b, respectively.

#### 3.1.1. Master Curves of Dynamic Modulus (|G*|)

**Figure 4:**
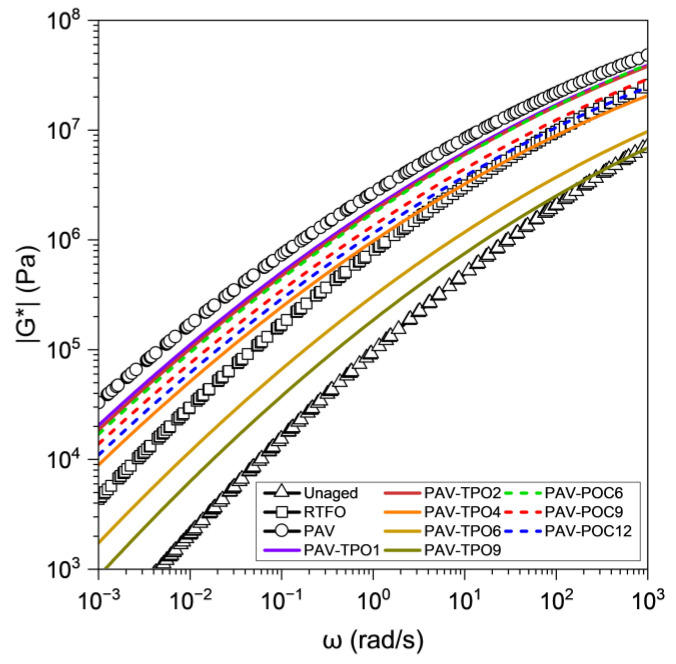
|G*| master curves at 25 °C for asphalt binder samples with, and without, additives.

The data collected at different temperatures can be shifted to a reference temperature, forming a continuous curve using time-temperature superposition. The continuous curve, known as the master curve, allows for the description of the material’s behavior over longer timescales than the experimental data alone can provide. Figure 4 shows the master curves of |G*| at 25 °C for the unaged, RTFO, PAV and PAV containing different doses of TPO and POC additives.

**Figure 5:**
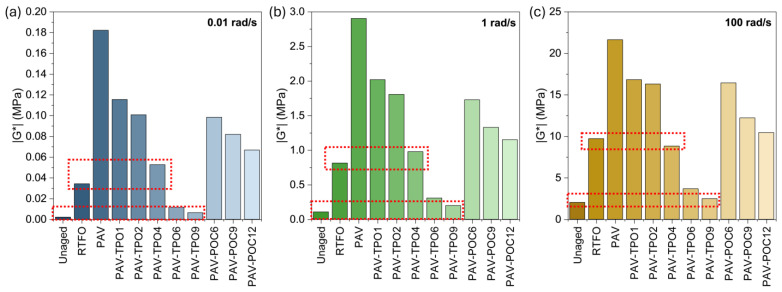
|G*| values from master curves at (**a**) low, (**b**) intermediate, and (**c**) high frequencies. Lower red line indicates similarity between Unaged and PAV-TPO9. Upper red line indicates similarity between RTFO and PAV-TPO4.

Additionally, Figure 5 shows the |G*| values at three representative frequencies for the low (0.001 to 0.1 rad/s), intermediate (0.1 to 10 rad/s) and high (10 to 1000 rad/s) frequency ranges. The representative frequencies were selected in the middle of each range: 0.01, 1 and 100 rad/s, respectively for the low, intermediate and high frequencies of the master curve. The time-temperature superposition principle also enables the interpretation of results across different temperature ranges by correlating them with various frequency ranges. High, intermediate and low frequencies represent the low, intermediate and high temperatures, respectively [40].

The |G*| curves for unaged, RTFO and PAV in Figure 4 allow for observing the effect of aging on the stiffness of the asphalt binder. The unaged curve exhibits the lowest |G*| values across the entire frequency range, indicating the softer characteristics of the unaged binder. The RTFO curve demonstrates a significant increase in |G*| after the binder undergoes short-term aging in the RTFO oven. Figure 5 shows the RTFO increase in |G*| to be between 5 times (high frequency) and 15 times (low frequency), when compared to the unaged binder, highlighting the significant effect of aging on the material’s stiffness during mix production and pavement construction. Similarly, the PAV curve shows further increases in |G*| after long-term aging in the PAV oven, with stiffness increasing between 2 and 5 times, compared with the RTFO short-term aged binder, underscoring the significant effect of aging on the binder’s stiffness throughout the pavement’s service life.

After the addition of TPO to the PAV-aged binder, the |G*| undergoes a reduction, regardless of the format of the rejuvenator (liquid TPO or encapsulated POC). Furthermore, with increasing TPO dosage, the stiffness of the material decreased, attributed to the softening effect of the rejuvenator, suggesting a potential chemical-level alteration (to be discussed in the following section). Figure 5 shows that the incorporation of 4% TPO into the PAV binder (PAV-TPO4) yielded stiffnesses comparable to the RTFO sample, with a more pronounced effect at higher frequencies, as pointed out by the top red box on each graph. Furthermore, Figure 5 illustrates that increasing the TPO dosage to 9% restores stiffness to a level comparable to the unaged binder, as pointed out by the lower red box on each graph. The softening effect is more significant at higher frequencies than at lower frequencies, since at higher frequencies the PAV-TPO9 curve is closer to the unaged curve than at low frequencies.

The addition of POC also resulted in a softening of the PAV binder to levels that suggest its rejuvenation. The alginate-based capsule matrix can be considered inert in this rejuvenation process, as the physicochemical interaction between the polysaccharide surface and the binder matrix is negligible [41]. In other words, the capsule material alone should not cause any softening of the asphalt binder. Thus, this observation can be attributed to the release of TPO from the capsules into the asphalt binder during mixing or DSR testing. Since TPO is a low-viscosity, maltene-rich oil, its incorporation into the binder reduces viscosity through dilution and decreased intermolecular friction—a well-known effect also reported in classical rheological studies of bitumen modified with extender oils and rejuvenators [6,11]. Higher concentrations of capsules resulted in greater softening. This softening effect was more pronounced at high frequencies than at low frequencies, similar to the behavior observed with the TPO samples.

The main conclusion of this section is that adding TPO or POC to the PAV-aged binder rejuvenates the material by reducing its dynamic modulus, with higher dosages leading to a more significant reduction in stiffness. In new asphalt pavement construction, the softer, rejuvenated binder is expected to exhibit significantly reduced brittleness and enhanced resistance to cracking compared to the PAV-aged binder.

#### 3.1.2. Master Curves of Phase Angle (δ)

**Figure 6:**
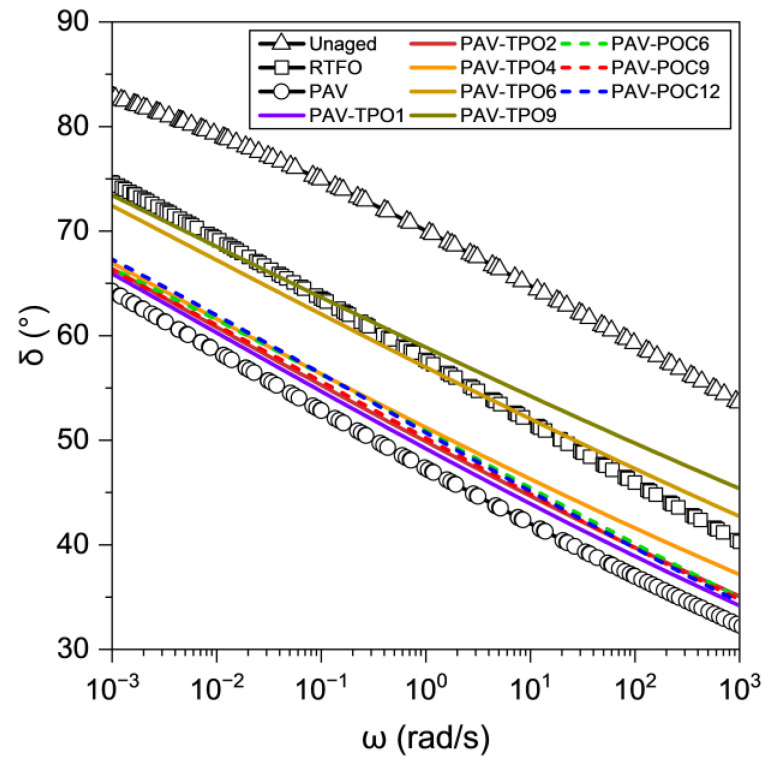
δ Master curves at 25 °C for asphalt binder samples with, and without, additives.

**Figure 7:**
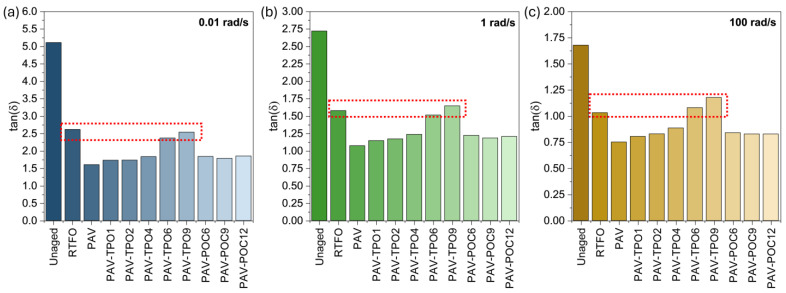
Tan(δ) values from master curves at (**a**) low, (**b**) intermediate, and (**c**) high frequencies. Red line indicates similarity between RTFO and PAV-TPO6/PAV-TPO9.

The time temperature superposition principle was also used to obtain the master curves for δ. Figure 6 shows the master curves of δ at 25 °C for all materials tested. Figure 7 shows the tan(δ) values at low (0.01 rad/s), intermediate (1 rad/s) and high (100 rad/s) frequencies. Tan(δ) allows for identifying the transition between the viscous-dominated and elastic-dominated responses, which occurs when tan(δ) equals 1.

The δ curves for unaged, RTFO, and PAV in Figure 6 illustrate the effect of aging on the viscoelasticity of the asphalt binder. The unaged curve shows the highest δ values across the entire frequency range, indicating a more viscous response in the unaged binder. After short-term aging in the RTFO oven, the RTFO curve reveals a significant decrease in δ, with a reduction between 10% (low frequency) and 20% (high frequency) compared to the unaged binder, highlighting the negative impact of short-term aging on the binder’s ability to undergo viscous flow. Similarly, the PAV curve indicates an additional δ reduction of 10% to 20% compared to the RTFO-aged binder.

The results show that adding TPO increases the δ of the PAV-aged binder, helping to restore the viscous component that allows for stress relief and healing of microcracks [42]. Higher amounts of TPO lead to greater increases in δ. However, the rejuvenator’s effect on δ is less pronounced than on |G*|. To achieve tan(δ) values similar to those of the RTFO short-term aged binder, 6% to 9% of TPO had to be added to the PAV binder, as pointed out by the red boxes on each graph. These values are high, compared to just 4% needed for the same effect on |G*|. None of the TPO percentages used were sufficient to restore the viscous response to levels comparable to the unaged binder, suggesting that higher percentages of TPO would be needed for this purpose. The addition of POC also increased the viscous component of the PAV-aged binder, by releasing TPO from the capsules into the asphalt binder during the mixing process or during DSR testing.

The results presented in this section demonstrate that adding TPO or POC to the PAV-aged binder increases δ, partially restoring the binder’s viscous response and enhancing its ability to relieve stress and heal microcracks. For the same dosages, the rejuvenating effect observed on the viscous component (δ) of the PAV-aged binder was not as significant as the one achieved on its stiffness (G*).

#### 3.1.3. Amount of Rejuvenator Released from Capsules During Mixing and Testing

**Figure 8:**
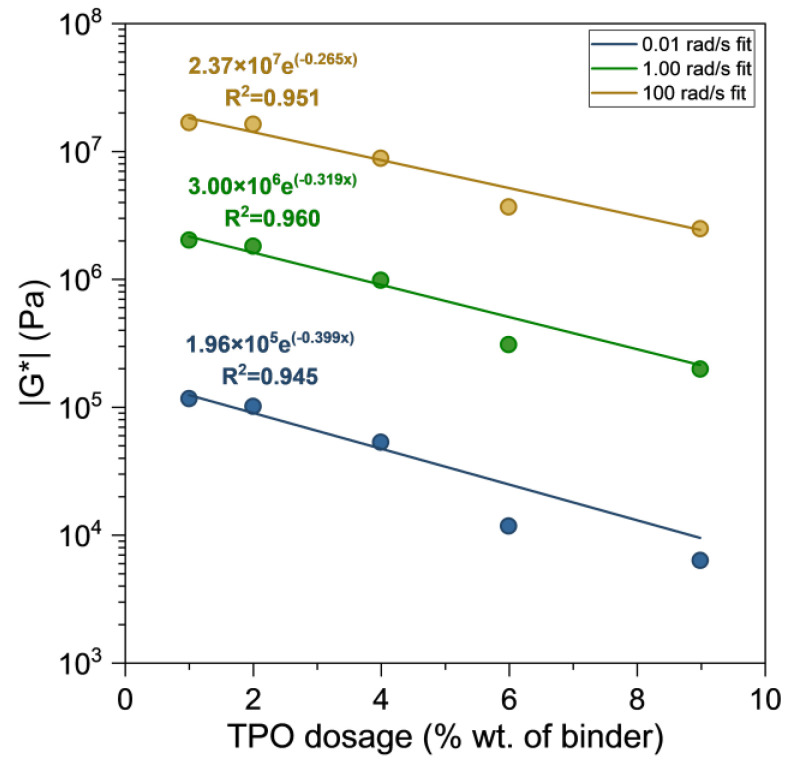
G* versus the amount of rejuvenator for TPO samples.

By comparing the rejuvenating effects of the TPO and POC samples, a rough estimate of the amount of TPO released from the capsules during the mixing and testing period can be obtained. Figure 8 shows the softening effect on |G*|, as a function of rejuvenator dosage for the liquid TPO samples at low, intermediate and high frequencies. The data were well represented by exponential regression models, which are also presented in Figure 8.

The capsules were designed with a container-to-liquid weight ratio of 1:5. Consequently, sample PAV-POC6 contained 1% polymeric container and 5% liquid TPO, PAV-POC9 contained 1.5% container and 7.5% liquid, and PAV-POC12 contained 2% multi-cavity container and 10% liquid TPO. The amount of TPO released from these encapsulated samples was estimated by substituting their measured |G| values into the regression equations obtained for the liquid TPO samples at the corresponding frequencies. The estimated released contents are presented in the three Column A entries of Table 2, corresponding to the PAV-POC6, PAV-POC9, and PAV-POC12 samples.

Table 2 shows that the fraction of TPO released from the POC during the mixing and testing process ranged between 0.27 and 0.35, as indicated by the A/B ratios. This finding suggests that the breakdown-diffusion-healing mechanisms of encapsulated rejuvenators result in slower self-healing kinetics compared to direct liquid TPO. However, this slower release is not an operational disadvantage, as asphalt containing encapsulated rejuvenators is specifically designed for a sustained, long-term release of self-healing properties. According to Norambuena-Contreras et al., once the polynuclear capsules are activated within the asphalt mixture, the release and diffusion of the encapsulated rejuvenating agent result in a softening effect on the bitumen [43]. This softening enables the bitumen to flow into the open crack, effectively sealing it.

#### 3.1.4. Rheological Properties and Rejuvenator Applications

**Figure 9:**
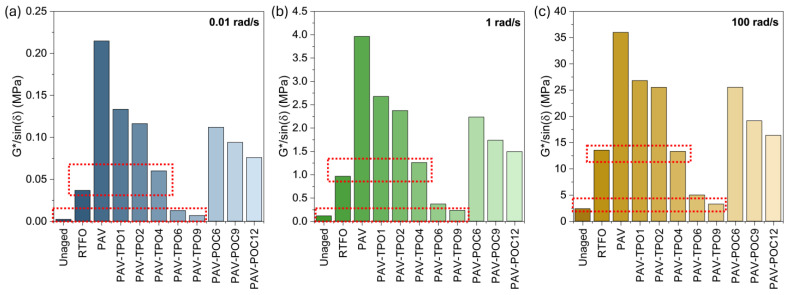
G*/sinδ parameter at (**a**) low, (**b**) intermediate, and (**c**) high frequencies. Lower red line indicates similarity between Unaged and PAV-TPO9. Upper red line indicates similarity between RTFO and PAV-TPO4.

The amount of rejuvenator mixed with aged asphalt binder varies by application, as outlined in Section 1. For recycling RAP into new asphalt, the binder must be rejuvenated to near-unaged conditions due to primary ageing during production. Encapsulated rejuvenator only restores the binder to a primary-aged state, as it releases post-construction, after primary ageing. Excessive rejuvenator must be avoided to prevent over-softening, which increases the risk of pavement deformation. For this reason, some authors recommend using rutting parameters to limit the amount of rejuvenator that should be applied [24,25]. In this article, the Superpave rutting parameter G*/sinδ (ASTM D6373-21A) will be used for this purpose, since it captures in one value the contribution of the rejuvenating effect in both rheological properties G* and δ [31]. Figure 9 shows the G*/sinδ parameter for the tested samples at (a) low, (b) intermediate and (c) high frequencies.

Since G*/sinδ represents a minimum threshold value that minimizes the risks of permanent deformation in the pavement, the G*/sinδ values of the unaged and RTFO binders will be used as the threshold for limiting the amount of TPO to be applied, depending on the desired application. Figure 9 shows that using 9% of TPO in the PAV binder would bring its G*/sinδ to comparable levels, but not below, the value measured in the unaged binder. This means that 9% TPO would be an appropriate dosage for rejuvenating RAP with binder in PAV state, making it viable for recycling into new asphalt pavement.

Figure 8 shows that adding 4% TPO to the PAV binder raises the G*/sinδ parameter to values comparable with those observed under RTFO conditions. At low and intermediate frequencies, the values remain slightly higher than RTFO, while at high frequencies they are slightly lower. This minor deviation at high frequencies is not critical for rutting performance, because at low temperatures (high frequencies) the high binder stiffness already prevents permanent deformation [3]. Based on this, 4% TPO can be considered an appropriate dosage for encapsulated applications. For such applications, the effective capsule dosage depends on both the container-to-liquid ratio and the release kinetics of the rejuvenator. In this study, a 12% POC dosage was found suitable, as it released enough TPO during mixing to reach RTFO-level performance without reducing G*/sinδ below the threshold. The remaining TPO content is expected to be released gradually during the pavement service life, supporting the long-term self-healing process described previously.

### 3.2. FTIR-ATR Characterization of the Asphalt Binders

**Figure 10:**
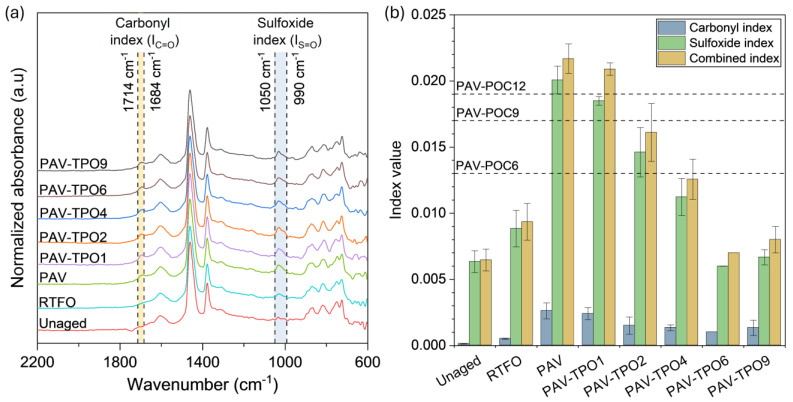
(**a**) FTIR-ATR characterization results, and (**b**) carbonyl, sulfoxide, and combined indices for the unaged, RTFO, PAV, PAV samples with TPO addition (1%, 2%, 4%, 6%, and 9%) and combined index of PAV samples with POC addition (6%, 9, and 12%); see the dotted lines.

The FTIR-ATR spectra of unaged, RTFO, PAV and PAV with TPO and POCs are shown in Figure 10a. The interpretation of the spectra was carried out by using the signal association depicted in Table 3. The spectral window shown in Figure 10a was truncated to emphasize regions where the rejuvenator is expected to have a significant chemical effect. All binders, whether virgin or aged, exhibited characteristic absorption bands of aromatic groups at 748, 810, 869, and 1600 cm^−1^.

Furthermore, they displayed absorption bands at 720, 1375, and 1455 cm^−1^, typically attributed to alkyl functional groups. Moreover, the presence of absorption bands around 1027 cm^−1^ and at 1710 cm^−1^ (highlighted in Figure 10a) indicates the existence of S=O and C=O bending vibrations, respectively. These groups are often linked with the degree of aging in asphalt binders, serving as chemical data-driven descriptors to assess the rejuvenating effect of TPO and POCs on the PAV binder.

The addition of TPO into PAV asphalt binder resulted in a chemical restoration of the binder, as evidenced by the significant decrease in the $I_{C=O}$, $I_{S=O}$, and $I_{\mathrm{Comb}}$ indexes shown in Figure 10b. Interestingly, between 6% and 9% of TPO, there appears to be a plateau in these indexes, which could be attributed to the detection of S-containing groups originating from the TPO. Although significant concentrations of S-containing species were not detected during GC-MS analysis, these compounds may be present in the heavier fraction of the TPO (which is undetectable by GC-MS), considering that waste tires typically contain around 1.8% elemental sulfur. The rejuvenation induced by TPO in the PAV-aged binder suggests that the TPO dosage required to revert from PAV aging to unaged conditions, as required for preparing RAP mixtures, ranges between 6% and 9%. To achieve the RTFO condition, as for encapsulated applications, 4% to 6% of TPO would be needed. This result coincides with the dosage estimated from the rheological analysis.

To confirm the previous analysis, statistically significant differences for the $I_{\mathrm{Comb}}$ were determined by performing one-way Analysis of Variance (ANOVA) and Tukey pairwise mean comparison. Taking as reference the unaged, RTFO, and PAV samples, the ANOVA revealed similar $I_{\mathrm{Comb}}$ when comparing: (i) unaged with PAV-TPO6 and TPO9, (ii) RTFO with PAV-TPO6 and TPO9, and (iii) PAV with PAV-TPO1. The $I_{\mathrm{Comb}}$ for PAV-TPO2 and TPO4 were statistically different between them and the rest of samples. Such results allow us to conclude an effective rejuvenation of the PAV-aged condition starting from 2% of TPO. Between 2 and 4% of TPO produce a rejuvenation between PAV and RTFO, and between 6% and 9% of TPO results in a bitumen rejuvenation between RTFO and unaged.

When adding POCs to the PAV in concentrations of 6%, 9%, and 12%, the $I_{\mathrm{Comb}}$ decreased proportionally to the capsule dosage. This behavior cannot be solely attributed to the mass effect of the liquid, as we initially expected a direct correlation between the rejuvenation action and the amount of capsules added. Considering the approximately 30% difference in $I_{\mathrm{Comb}}$ between PAV-POC6 and PAV-POC12, it is conceivable that the diffusion of the liquid and the mixing conditions may be influencing the kinetics of rejuvenation. The kinetics of this process are influenced by various factors such as mixing conditions (mixing speed and rejuvenator dosage), temperature, concentration of rejuvenating species, and contact time. Therefore, an operational distinction exists between the direct application of TPO and POC, which should be carefully considered in further analysis. Despite the reduction in the index, the restoration facilitated by the capsules induced a rejuvenating effect comparable to that of liquid TPO at doses ranging from 1% to 4%, as also observed in the rejuvenation of the rheological parameters.

## 4. Conclusions

This study, for the first time, evaluated the rejuvenating effects of TPOs on binders with varying aging levels, comparing two application types: liquid TPO and encapsulated POCs. Binder rheological properties (|G*|, δ) and chemical composition were assessed through FTIR analysis. Based on the results, the following conclusions were drawn:

- **Rejuvenation of aged asphalt binder:** Waste tire pyrolytic oil (TPO) effectively rejuvenates aged asphalt binders by reducing stiffness (G*), increasing viscous response (δ), and lowering carbonyl and sulfoxide aging indices of long-term aged binders (PAV). According to FTIR results, carbonyl and sulfoxide indices decreased progressively with TPO dosage, while rheological analysis showed that 4% TPO brought |G*| of the PAV binder to levels comparable to RTFO, and 9% TPO restored stiffness close to the unaged binder. Higher TPO dosages enhance these effects. Additionally, capsules (POC) containing TPO help rejuvenate PAV-aged binders through partial TPO release into the binder.
- **Potential for recycling reclaimed asphalt pavement (RAP):** The required amount of TPO to rejuvenate a PAV-aged binder to a state similar to its unaged condition was found to be 9% by binder weight. At this dosage, G/sinδ reached values comparable to the unaged binder. This dosage is sufficient for rejuvenating PAV-aged binders in RAP mixtures for reuse in new pavement construction. The dosage was determined by using the rutting parameter (G*/sinδ) and confirmed by FTIR results.
- **Amount of rejuvenator released from capsules:** It was estimated that capsules (POC) released 27% to 35% of their TPO content during mixing and DSR testing. This conclusion was based on comparisons of G*, δ, and FTIR results between POC capsules and direct TPO applications. For instance, 12% POC showed a softening effect similar to 4% TPO addition, due to the partial rejuvenator released. The capsules are designed for slow release, enabling long-term self-healing in pavements as the remaining TPO is gradually released over time.
- **Encapsulated applications:** For asphalt pavements with binders in PAV-aging conditions, the amount of TPO released from capsules should not exceed 4% by binder weight within a short period, assuming ideal diffusion. Higher release levels could cause excessive softening, increasing the risk of rutting, as indicated by the G*/sinδ parameter. The required capsule amount depends on their TPO content and release rate over time. In this study, a 12% POC dosage successfully released 4% TPO during mixing and testing of binders, achieving rheological properties comparable to the RTFO-aged condition.

## 5. Patents

A portion of this study is currently under patent evaluation. Patent application No. 202402303 was submitted to the National Institute of Industrial Property (INAPI), Chile.

## Figures and Tables

**Table 1:** Main properties of asphalt binder PG64-22.

Property	Value	Standard
Density	1.04 g/cm^3^	ASTM D70/D70M-21
Penetration @ 25 °C	53 dmm	ASTM D946/D946M-20
Softening Point	52.2 °C	ASTM D36/D36M-12
G*/sin(δ) @ 64 °C (Unaged)	>1 kPa	ASTM D6373-21A
G*/sin(δ) @ 64 °C (RTFO-aged)	>2.2 kPa	ASTM D6373-21A
G*·sin(δ) @ 25 °C (PAV-aged)	5976	ASTM D6373-21A
S @-12 °C	222 MPa	ASTM D6373-21A
m @-12 °C	0.36	ASTM D6373-21A

**Table 2:** TPO released from capsules.

ω [rad/s]	PAV-POC6			PAV-POC9			PAV-POC12		
	A: TPO Released (*)	B: Total TPO Content	A/B: Fraction Released	A: TPO Released (*)	B: Total TPO Content	A/B: Fraction Released	A: TPO Released (*)	B: Total TPO Content	A/B: Fraction Released
0.01	1.73%	5%	0.35	2.19%	7.5%	0.29	2.70%	10%	0.27
1	1.73%	5%	0.35	2.55%	7.5%	0.34	3.01%	10%	0.30
100	1.38%	5%	0.28	2.50%	7.5%	0.33	3.09%	10%	0.31

(*) The values were calculated from the regression shown in Figure 8.

**Table 3:** FTIR functional groups in the bitumen.

Wavelength (cm^−1^)	Assigned Vibration (*)	Functional Group
720	*ρ(CH_2_)_n_*	*Alkyls*
748	*γCH_aro_*	*Aromatic*
810	*γCH_aro_*	*Aromatic*
869	*γCH_aro_*	*Aromatic*
-	*δ(CH)*	*Alkene*
1027	*νS=O*	*Sulfoxides*
1160	*νSO_2_ (in* *-* *phase)*	*Sulfones*
1375	*δ_s_CH_2_/CH_3_*	*Alkyls*
1455	*δ_as_CH_2_/CH_3_*	*Alkyls*
1600	*νC=C*	*Aromatic*
1710	*νC=O*	*Ketone*

(*) Signals attribution was performed by following the criteria of Ma et al. [44,45].

## Data Availability

The raw data supporting the conclusions of this study are provided within the article and its Supplementary Materials.

## Supplementary Materials

The following supporting information can be downloaded at: https://www.mdpi.com/article/10.3390/polym17182449/s1. Table S1. Results for the dynamic shear modulus (|G*|) at the different testing temperatures and frequencies. Table S2. Results for the phase angle (δ) at the different testing temperatures and frequencies. Table S3. Average results and standard deviation (SD) of the Carbonyl ($I_{C=O}$), Sulfoxide ($I_{S=O}$), and Combined ($I_{\mathrm{Comb}}$) indices. Table S4. Analysis of Variance (ANOVA) for the combined indices of each bitumen sample. Table S5. Tukey pairwise means comparison for the combined indices of each bitumen sample.

## Abbreviations

The following abbreviations are used in this manuscript:

TPO	Tire Pyrolytic Oil
POC	Pyrolytic Oil Capsules
RTFO	Rolling Thin Film Oven
PAV	Pressure Aging Vessel
RAP	Reclaimed Asphalt Pavements
MD	Molecular dynamics
FTIR	Fourier Transform Infrared spectroscopy
GC-MS	Gas Chromatography coupled with Mass Spectrometry
ASTM	American Society for Testing and Materials
DSR	Dynamic Shear Rheometer
ANID	Chilean National Agency for Research and Development
UTFSM	Universidad Técnica Federico Santa María
