# Supplementary material for: Rheological and Chemical Effects of Waste Tire Pyrolytic Oil and Its Encapsulation as Rejuvenators on Asphalt Binders
Source: Polymers (Basel). 2025 Sep 10;17(18):2449. doi: 10.3390/polym17182449 (PMC12473857; doi:10.3390/polym17182449)
Supplement: Supplementary file 1 [file polymers-17-02449-s001.zip › polymers-3847853-supplementary.pdf]

Supplementary data

Table S1. Results for the dynamic shear modulus (|G*|) at the different testing temperatures and frequencies.

	T=5°C	T=10°C	T=15°C	T=20°C	T=25°C	T=30°C	T=35°C	T=40°C	T=45°C	T=50°C	T=55°C	T=60°C	T=65°C	T=70°C	T=75°C
**ω [rad/s]**	**|G*| [Pa]**	**|G*| [Pa]**	**|G*| [Pa]**	**|G*| [Pa]**	**|G*| [Pa]**	**|G*| [Pa]**	**|G*| [Pa]**	**|G*| [Pa]**	**|G*| [Pa]**	**|G*| [Pa]**	**|G*| [Pa]**	**|G*| [Pa]**	**|G*| [Pa]**	**|G*| [Pa]**	**|G*| [Pa]**
**100**	7.2E+07	4.7E+07	2.9E+07	1.6E+07	8.7E+06	4.5E+06	2.2E+06	1.2E+06	6.7E+05	3.6E+05	2.0E+05	1.1E+05	6.0E+04	3.4E+04	1.9E+04
**61.1**	6.2E+07	4.0E+07	2.4E+07	1.3E+07	7.0E+06	3.5E+06	1.6E+06	8.5E+05	4.9E+05	2.6E+05	1.4E+05	7.6E+04	4.1E+04	2.3E+04	1.2E+04
**37.3**	5.4E+07	3.4E+07	2.0E+07	1.1E+07	5.5E+06	2.7E+06	1.2E+06	6.3E+05	3.5E+05	1.8E+05	9.8E+04	5.3E+04	2.8E+04	1.5E+04	8.2E+03
**22.8**	4.7E+07	2.9E+07	1.6E+07	8.7E+06	4.4E+06	2.1E+06	9.3E+05	4.6E+05	2.5E+05	1.3E+05	6.8E+04	3.6E+04	1.9E+04	1.0E+04	5.4E+03
**13.9**	4.0E+07	2.4E+07	1.3E+07	7.0E+06	3.4E+06	1.6E+06	6.9E+05	3.4E+05	1.8E+05	9.1E+04	4.7E+04	2.5E+04	1.3E+04	6.6E+03	3.5E+03
**8.48**	3.4E+07	2.0E+07	1.1E+07	5.6E+06	2.7E+06	1.2E+06	5.1E+05	2.4E+05	1.3E+05	6.4E+04	3.2E+04	1.7E+04	8.4E+03	4.4E+03	2.3E+03
**5.18**	2.9E+07	1.7E+07	9.0E+06	4.5E+06	2.1E+06	9.0E+05	3.8E+05	1.8E+05	9.0E+04	4.4E+04	2.2E+04	1.1E+04	5.6E+03	2.8E+03	1.4E+03
**3.16**	2.5E+07	1.4E+07	7.3E+06	3.5E+06	1.6E+06	6.8E+05	2.8E+05	1.3E+05	6.3E+04	3.1E+04	1.5E+04	7.5E+03	3.7E+03	1.8E+03	9.2E+02
**1.93**	2.1E+07	1.2E+07	6.0E+06	2.8E+06	1.2E+06	5.1E+05	2.0E+05	9.1E+04	4.4E+04	2.1E+04	1.0E+04	4.9E+03	2.4E+03	1.2E+03	5.8E+02
**1.18**	1.8E+07	9.7E+06	4.8E+06	2.2E+06	9.5E+05	3.8E+05	1.5E+05	6.5E+04	3.1E+04	1.4E+04	6.8E+03	3.2E+03	1.5E+03	7.4E+02	3.6E+02
**0.72**	1.5E+07	8.0E+06	3.9E+06	1.7E+06	7.3E+05	2.9E+05	1.1E+05	4.6E+04	2.1E+04	9.8E+03	4.5E+03	2.1E+03	9.8E+02	4.7E+02	2.3E+02
**0.439**	1.3E+07	6.6E+06	3.1E+06	1.4E+06	5.6E+05	2.1E+05	7.8E+04	3.3E+04	1.5E+04	6.5E+03	3.0E+03	1.4E+03	6.2E+02	2.9E+02	1.4E+02
**0.268**	1.1E+07	5.5E+06	2.5E+06	1.1E+06	4.3E+05	1.6E+05	5.6E+04	2.3E+04	9.9E+03	4.3E+03	1.9E+03	8.6E+02	3.9E+02	1.8E+02	8.7E+01
**0.164**	9.1E+06	4.5E+06	2.0E+06	8.4E+05	3.3E+05	1.2E+05	4.1E+04	1.6E+04	6.7E+03	2.9E+03	1.2E+03	5.5E+02	2.4E+02	1.1E+02	5.3E+01
**0.1**	7.8E+06	3.8E+06	1.6E+06	6.6E+05	2.5E+05	8.8E+04	2.9E+04	1.1E+04	4.5E+03	1.9E+03	8.0E+02	3.4E+02	1.5E+02	6.9E+01	3.3E+01

Table S2. Results for the phase angle (δ) at the different testing temperatures and frequencies.

	T=5°C	T=10°C	T=15°C	T=20°C	T=25°C	T=30°C	T=35°C	T=40°C	T=45°C	T=50°C	T=55°C	T=60°C	T=65°C	T=70°C	T=75°C
**ω [rad/s]**	**δ [°]**	**δ [°]**	**δ [°]**	**δ [°]**	**δ [°]**	**δ [°]**	**δ [°]**	**δ [°]**	**δ [°]**	**δ [°]**	**δ [°]**	**δ [°]**	**δ [°]**	**δ [°]**	**δ [°]**
**100**	27.03	30.23	33.79	37.6	41.82	46.43	51.05	54.65	58.58	61.44	64.1	66.65	69.05	71.51	74.06
**61.1**	27.98	31.18	34.74	38.92	42.91	47.54	52.12	55.7	59.46	62.28	64.93	67.51	70.07	72.71	75.27
**37.3**	28.87	32.07	35.63	39.58	44.12	48.58	53.11	56.62	60.32	63.12	65.79	68.43	71.36	73.89	76.38
**22.8**	29.74	32.94	36.54	40.57	45.02	49.62	54.08	57.54	61.18	63.99	66.71	69.44	72.22	74.96	77.64
**13.9**	30.6	33.81	37.47	41.57	46.03	50.63	55.05	58.46	62.06	64.91	67.71	70.55	73.46	76.29	78.97
**8.48**	31.46	34.71	38.43	42.62	47.09	51.63	56.01	59.39	62.98	65.89	68.8	71.77	74.8	77.68	80.3
**5.18**	32.35	35.64	39.44	43.69	48.16	52.65	56.96	60.31	63.94	66.95	69.98	73.1	76.2	79.09	81.62
**3.16**	33.25	36.59	40.47	44.76	49.23	53.67	57.91	61.3	64.96	68.12	71.3	74.54	77.65	80.45	82.86
**1.93**	34.18	37.59	41.54	45.89	50.3	54.63	58.92	62.28	66.05	69.36	72.7	75.99	79.14	81.82	83.95
**1.18**	35.12	38.61	42.64	46.97	51.39	55.69	59.86	63.37	67.27	70.73	74.17	77.54	80.48	83.06	85.05
**0.72**	36.09	39.68	43.79	48.13	52.48	56.64	60.93	64.42	68.48	72.12	75.68	78.95	81.9	84.22	85.9
**0.439**	37.07	40.71	44.87	49.3	53.45	57.63	62.06	65.74	69.98	73.77	77.37	80.54	83.24	85.22	86.61
**0.268**	38.04	41.77	45.97	50.43	54.44	58.53	63.19	67.06	71.47	75.4	78.96	81.94	84.41	86.12	87.17
**0.164**	38.98	42.9	47.16	51.45	55.55	59.46	64.28	68.3	72.85	76.84	80.33	83.05	85.34	86.91	87.77
**0.1**	39.79	43.89	48.2	52.29	56.47	60.55	65.23	69.99	74.59	78.58	81.82	84.4	86.06	87.32	88.27

Table S3. Average results and standard deviation (SD) of the Carbonyl ($I_{C=O}$), Sulfoxide ($I_{S=O}$), and Combined (I_{Comb}) indices.

	$I_{C=O}$	SD	$I_{S=O}$	SD	I_{Comb}	SD
**Unaged**	1.28E-04	1.08E-05	6.33E-03	8.25E-04	6.46E-03	8.33E-04
**RTFO**	5.10E-04	5.06E-05	8.84E-03	1.37E-03	9.35E-03	1.38E-03
**PAV**	2.62E-03	5.97E-04	2.01E-02	1.05E-03	2.17E-02	1.11E-03
**PAV-TPO1**	2.40E-03	4.55E-04	1.85E-02	3.28E-04	2.09E-02	4.72E-04
**PAV-TPO2**	1.50E-03	6.56E-04	1.46E-02	1.87E-03	1.61E-02	2.19E-03
**PAV-TPO4**	1.34E-03	2.16E-04	1.12E-02	1.41E-03	1.26E-02	1.52E-03
**PAV-TPO6**	1.00E-03	0.00E+00	6.00E-03	1.06E-18	7.00E-03	0.00E+00
**PAV-TPO9**	1.33E-03	5.77E-04	6.67E-03	5.77E-04	8.00E-03	1.00E-03

Table S4. Analysis of Variance (ANOVA) for the combined indices of each bitumen sample.

	DF	Sum of Squares	Mean Square	F Value	Prob>F
**Model**	7	0.00132	1.89E-04	99.23843	<0.0001
**Error**	32	6.09E-05	1.90E-06		
**Total**	39	0.00138			

Table S5. Tukey pairwise means comparison for the combined indices of each bitumen sample.

**Sample**	**Mean**	**Groups**				
PAV	0.02169	A				
PAV-TPO1	0.0209	A				
PAV-TPO2	0.01611		B			
PAV-TPO4	0.01257			C		
RTFO	0.00934				D	
PAV-TPO9	0.008				D	E
PAV-TPO6	0.007				D	E
Unaged	0.00646					E
